# Duplication and positive selection among hominin-specific *PRAME *genes

**DOI:** 10.1186/1471-2164-6-120

**Published:** 2005-09-13

**Authors:** Zoë Birtle, Leo Goodstadt, Chris Ponting

**Affiliations:** 1 MRC Functional Genetics Unit, University of Oxford, Department of Human Anatomy and Genetics, South Parks Road, Oxford OX1 3QX UK

## Abstract

**Background:**

The physiological and phenotypic differences between human and chimpanzee are largely specified by our genomic differences. We have been particularly interested in recent duplications in the human genome as examples of relatively large-scale changes to our genome. We performed an in-depth evolutionary analysis of a region of chromosome 1, which is copy number polymorphic among humans, and that contains at least 32 *PRAME *(Preferentially expressed antigen of melanoma) genes and pseudogenes. *PRAME*-like genes are expressed in the testis and in a large number of tumours, and are thought to possess roles in spermatogenesis and oogenesis.

**Results:**

Using nucleotide substitution rate estimates for exons and introns, we show that two large segmental duplications, of six and seven human *PRAME *genes respectively, occurred in the last 3 million years. These duplicated genes are thus hominin-specific, having arisen in our genome since the divergence from chimpanzee. This cluster of *PRAME *genes appears to have arisen initially from a translocation approximately 95–85 million years ago. We identified multiple sites within human or mouse *PRAME *sequences which exhibit strong evidence of positive selection. These form a pronounced cluster on one face of the predicted PRAME protein structure.

**Conclusion:**

We predict that *PRAME *genes evolved adaptively due to strong competition between rapidly-dividing cells during spermatogenesis and oogenesis. We suggest that as *PRAME *gene copy number is polymorphic among individuals, positive selection of *PRAME *alleles may still prevail within the human population.

## Background

Humans and chimpanzees shared a common ancestor approximately 6–7 million years ago (MYA) [1]. Distinguishing characteristics, such as those relating to cognitive abilities, language, habitual upright gait, dentition, and susceptibility to malaria, are all assumed to be associated with genetic differences between these two species [2]. However, these phenotypic differences have been associated with specific human-chimpanzee sequence differences in fewer than a handful of cases. In humans, two coding changes in *FOXP2 *have been proposed to contribute to language acquisition [3], disruption in the *MYH16 *myosin heavy chain gene is proposed to have led to a reduction in masticatory muscles [4], and the pseudogenisation of a type I hair keratin has been associated with modifications in our hair keratin phenotype [5]

It is also unclear at which developmental stages, and in which tissues, such human-specific adaptations are first manifested. For example, the *abnormal spindle-like microcephaly-associated *(*ASPM*) gene has roles in mitosis, meiosis and cytokinesis, and is broadly expressed in many tissues. Yet it is a major determinant of cerebral cortical size [6] and has evolved adaptively in recent hominin evolution (reviewed in Ponting & Jackson (2005))[7]. As the signatures of recent adaptation are identified in the human genome it will be important to associate these DNA changes with molecular, cellular and physiological innovation.

With the sequencing of the human and chimpanzee genomes comes the possibility of discerning nucleotide changes that have been acquired adaptively and thus might be associated with physiological innovation [2]. Two factors, however, often confound such studies. First, the scarcity of substitutions (~1% [8,9]) between human and chimpanzee orthologous coding sequence provides insufficient statistical power to distinguish adaptive from neutral substitutions. Second, the chimpanzee genome has been sequenced only to low coverage (4-fold statistical coverage for panTro1; see [10]). As a result, the chimpanzee genome sequence contains many gaps, sequence inaccuracies and assembly artefacts. Such problems are exacerbated in regions containing identical or almost identical tandem segments which pose particular problems for both sequencing and assembly. Juxtaposed and virtually identical sequences are frequently represented either by only single versions in genome assemblies, or are absent altogether, thereby giving rise to gaps in the assembly.

By contrast, the human genome assembly is virtually complete and is accurate to approximately one error every 10^5 ^bases [11]. The human sequence's high statistical coverage gives rise to an assembly that is a mosaic of contributions from multiple individuals and thus does not represent any single genome. This mosaicism is less important for single nucleotide polymorphisms (SNPs) than it is for larger-scale polymorphisms, such as copy number polymorphisms (CNPs). This is because most SNPs are selectively neutral whereas the evidence suggests that this appears not to be the case for CNPs [12].

Identifying sequence changes that distinguish human and chimpanzee physiology, development and behaviour is a challenge not only because of errors and polymorphisms in genome assemblies, but also because the very types of sequence differences that contribute most to these characteristics remain ill-determined. The near-identity of human and chimpanzee orthologous coding sequence led to an initial suggestion that gene expression, rather than coding sequence change, is the major contributor to our differences [13]. However, it has become clear that most of the variations between human and chimpanzee in non-coding sequence are not adaptive either [14]. Identifying adaptive substitutions, whether in coding or non-coding sequence, remains a considerable problem.

Our approach has been not to investigate single nucleotide substitutions as potential substrates of adaptation. Rather, we wish to consider larger sequence differences between human and chimpanzee genomes, namely genes which have duplicated in a lineage-specific manner in the past 6–7 MY since the last common ancestor of the two species.

To this end, we recently determined the number of synonymous (silent) mutations per synonymous site (*K*_*S*_) between closely-related human genes and used this to predict the lineage-specificity of duplication events. We identified a relatively large fraction (5%) of human genes that have participated in duplication events since the last common ancestor with the rodents [11]. Gene pairs that together have accumulated few substitutions in synonymous sites (*K*_*S *_< 0.3) were suggested to be primate-specific. The vast majority of these paralogues pairs have accumulated even fewer silent substitutions (*K*_*S *_< 0.015), indicating that most human duplications occurred only in the past 3–4 million years, after the divergence of *Homo *and *Pan *lineages. It is not yet known whether these recent duplications in the mosaic human genome assembly are fixed in the human population, or instead represent CNPs, although the latter explanation now appears increasingly likely [15]. The functions of these recently-duplicated genes are not uniformly distributed. Genes involved in reproduction, chemosensation and host defense and immunity are over-represented [11]. 'Cancer Testis antigen' (CTA) genes, most of which are normally expressed in the testis but are also highly active in certain cancers [16], are another prominent category among the recently-duplicated human gene set. They are represented among a small number of gene families, including one whose founding member is *PRAME *('Preferentially expressed antigen of melanoma'), a human gene that is expressed highly in a large proportion of tumours [17,18]. In all cases, the physiological role of CTA genes in normal cells remains unclear, but their recent and extensive duplications are consistent with adaptive functions, such as chemosensation, immunity and reproduction [19]. Moreover, their specific expression in the testis and ovary argues for their involvement in the acquisition of innovative reproductive function during recent primate evolution.

CTA genes frequently have been duplicated on the human X chromosome [11,20] which might indicate a male selective advantage in possessing these genes. A mouse *PRAME*-like X-linked gene is known to be expressed specifically in spermatogonia, and may perform roles in the early stages of spermatogenesis [21]. Other members of this family are clustered together on an autosome, mouse chromosome 4. Because mammalian sex chromosomes undergo inactivation in late stages of spermatogenesis, it is possible that X-linked *PRAME *genes may play a part early in spermatogenesis, whereas the cluster of autosomal *PRAME *genes functions either in later stages, or in other tissues. Indeed, one autosomal mouse *PRAME*-like gene is known to be expressed in both oocytes and early cleavage-stage embryos [22].

Here we describe the extraordinary recent evolution of autosomal *PRAME*-like gene clusters on human and chimpanzee chromosomes 1, and mouse chromosome 4. We use a molecular clock, calibrated using synonymous or intronic nucleotide substitutions, to infer the recent origin of many of these *PRAME *genes. This is corroborated independently by comparison with available chimpanzee genomic sequence. Our analyses confirm that these human genes have duplicated unusually rapidly within the last 3 MY, with concomitant and substantial sequence diversification resulting from adaptive evolution. We predict that the differences between human and chimpanzee *PRAME *genes contributed to the functional divergence along the hominin lineage.

## Results

### Evolutionary survey of 7 human CT-Antigen gene families

We investigated whether rapidly-duplicating members of seven CTA families have experienced rapid sequence diversification as a result of adaptive evolution. Using ENSEMBL gene predictions, we initially used codeml [23] to predict sites in their amino acid alignments that have been subject to positive selection. Only 2 of the 7 families, namely the *PRAME *genes and SSX-like genes, were predicted to contain positively-selected sites (posterior probabilities > 0.9 for each of three model pairs (see Methods)). Because of its large size and because of the large number (23) of positively-selected sites found in an initial analysis (using the NCBI34 genome assembly (data not shown)), we decided to perform a more comprehensive analysis of *PRAME *genes and pseudogenes in human, chimpanzee and mouse genomes; the human SSX-like family contains only 7 members, for which 6 positively-selected sites were predicted (data not shown).

### Recent origin for the *PRAME *gene cluster

We then investigated whether *PRAME *genes, located between RefSeq genes *DHRS3 *and *T1A-2 *on HSA1, are present in orthologous locations in other vertebrates. Indeed, the mouse genome contains *PRAME*-like genes in its orthologous region [24], as does the rat genome. However, the orthologous regions of both dog and chicken genome assemblies possess no *PRAME *homologous genes, as determined by searches of these regions using TBlastn [25]. Moreover, this region of the dog genome assembly contains no clone gaps and insufficiently large (≥ 2.5 kb) fragment gaps to accommodate any missing dog *PRAME *genes. As humans and rodents shared a more recent common ancestor than either humans and dogs, or humans and chickens, it thus appears likely that one or more *PRAME *genes were translocated into this genomic location after the divergence with the Laurasiatherian lineage containing extant carnivores (approximately 95 MYA), but before the rodent-primate split (approximately 85 MYA) [26].

### Human, chimpanzee and mouse *PRAME *genes

**Figure 1:**
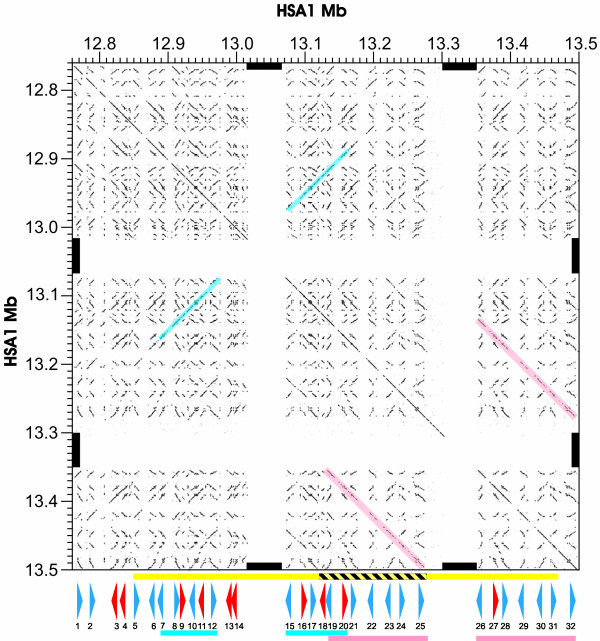
**Dot plot representation [63] of a 0.74 Mb region of human chromosome 1 (bases 1276000–1350000) annotated (below) according to the locations of *PRAME *genes (blue arrowheads) and pseudogenes (red arrowheads), approximately to scale. **Gene or pseudogene orientation is indicated by arrowhead direction. *PRAME *gene or pseudogene numbers are provided beneath the arrowheads. Single short diagonals represent alignments of two *PRAME *genes or pseudogenes. Gaps in the assembly (bases 13015219–13065218 and 13302469–13352468) are indicated, on the axes, by thick black bars. Two recent segmental duplications (*Homo_7–12 *and *15–20*, and *Homo_19–25 *and *26–32*; see text) are highlighted in blue and pink, respectively. Regions identified by Sebat et al. [12] or by Iafrate et al. [36], as being copy number polymorphic are indicated by a yellow, or a black-and-yellow-striped, bar, respectively.

Our comprehensive reprediction of human *PRAME *homologues from the 0.74 Mb region of HSA1 yielded a total of 22 *PRAME *genes and 10 pseudogenes (Figure 1). (These we number sequentially along the assembly, *Homo*_1, *Homo*_2, etc.) Each of these genes is approximately 3.0 kb long (average 3069 bases), contain three exons (labelled A, B and C) and two introns (*a *and *b*) both with consensus (GT-AG) splice sites. The translated protein is approximately 474 amino acids in length with the three exons having median lengths of 95, 193 and 186 amino acids.

Initial predictions of chimpanzee *PRAME *genes, from placed and unplaced PTR1 sequence and from unmapped assembled sequence, yielded 17 candidate genes. Careful inspection, however, revealed these gene predictions to be of poor quality with many predicted genes spanning suspiciously large (>> 3 kb) genomic distances and exhibiting regions of poor sequence similarity. We believe this is a result of the sequence incompleteness and the low (4-fold) statistical coverage of the chimpanzee genome sequence in the assembly. We thus instead resorted to independently predicting each of the three chimpanzee *PRAME *exons. This resulted in 16 exon A, 24 exon B and 19 exon C predictions. Several of these predictions appear to be identical and could thus be redundant. Adjacent exons and introns were assembled to give 12 putative chimpanzee *PRAME *genes and pseudogenes (labelled *Pan_1*, *Pan_2 *etc.), all of whose introns were confirmed to contain consensus (GT-AG) splice sites. Of these predictions, only 3 appear to be full length, with 3 exons and 2 introns lacking gaps. 30 predictions contain a single exon only. 7 of the 12 sequences contain 3 stop codons and 12 frameshifts, and so might be pseudogenes. (At suggested nucleotide substitution rate of 3 × 10^-4 ^and insertion/deletion error rates of approximately 2 × 10^-4 ^(Tarjei Mikkelsen, personal communication) we expect few if any of these disruptions to arise from sequencing errors (data not shown)).

We inferred relationships between chimpanzee exons and intron, and their human orthologous sequences, using phylogenetic trees (see below). This revealed both well assembled chimpanzee sequence, with consecutive exons and introns assigned to the same human orthologous gene, and poorly assembled sequences, manifested by short contigs, separated by gaps, in a disordered arrangement.

18 *PRAME*-like genes and 15 pseudogenes were predicted in the orthologous region of mouse chromosome 4. (These are numbered sequentially *Mus_1*, *Mus_2 *etc., in the same orientation as that used for the human and chimpanzee numbering scheme.) Of these 5 (*Mus_1*, *Mus_9*, *Mus_10*, *Mus_18 *and *Mus_30*) have previously been investigated by Dade et al. [24], who describe these as having roles in oogenesis.

### Local gene duplication

In order to visualise the chromosomal landscape of this region of HSA1, we compared its repeat-masked DNA sequence with itself using a dotplot representation (Figure 1). As befits tandemly-duplicated and highly similar sequence, a strong pattern of many diagonals was evident. Each short diagonal represents the DNA alignment of two *PRAME *genes or pseudogenes. The orientation of the diagonal indicates whether these two genes or pseudogenes are situated on the same, or else the opposite, strand. We observed two pairs of long diagonals (highlighted in colour in Figure 1) which represent two predicted events of segmental duplication (see below).

### Human and mouse *PRAME *genes are monophyletic

We then were able to exploit these gene predictions from human, chimpanzee and mouse to infer the genes' evolutionary relationships and the sequential order of gene duplications. At this stage, we do not rule out that paralogous sequences have been subject to recent inter-locus gene conversion [27,28] which may result in greater sequence similarity and, hence, an apparently more recent date of evolutionary divergence (see Discussion). Dendrograms were constructed from two types of quasi-neutral nucleotide substitution rates: *K*_*S *_values, either for single coding exons, or for complete coding sequence, and *K*_*I *_values, defined as the numbers of nucleotide substitutions per site within intronic sequence.

**Figure 2:**
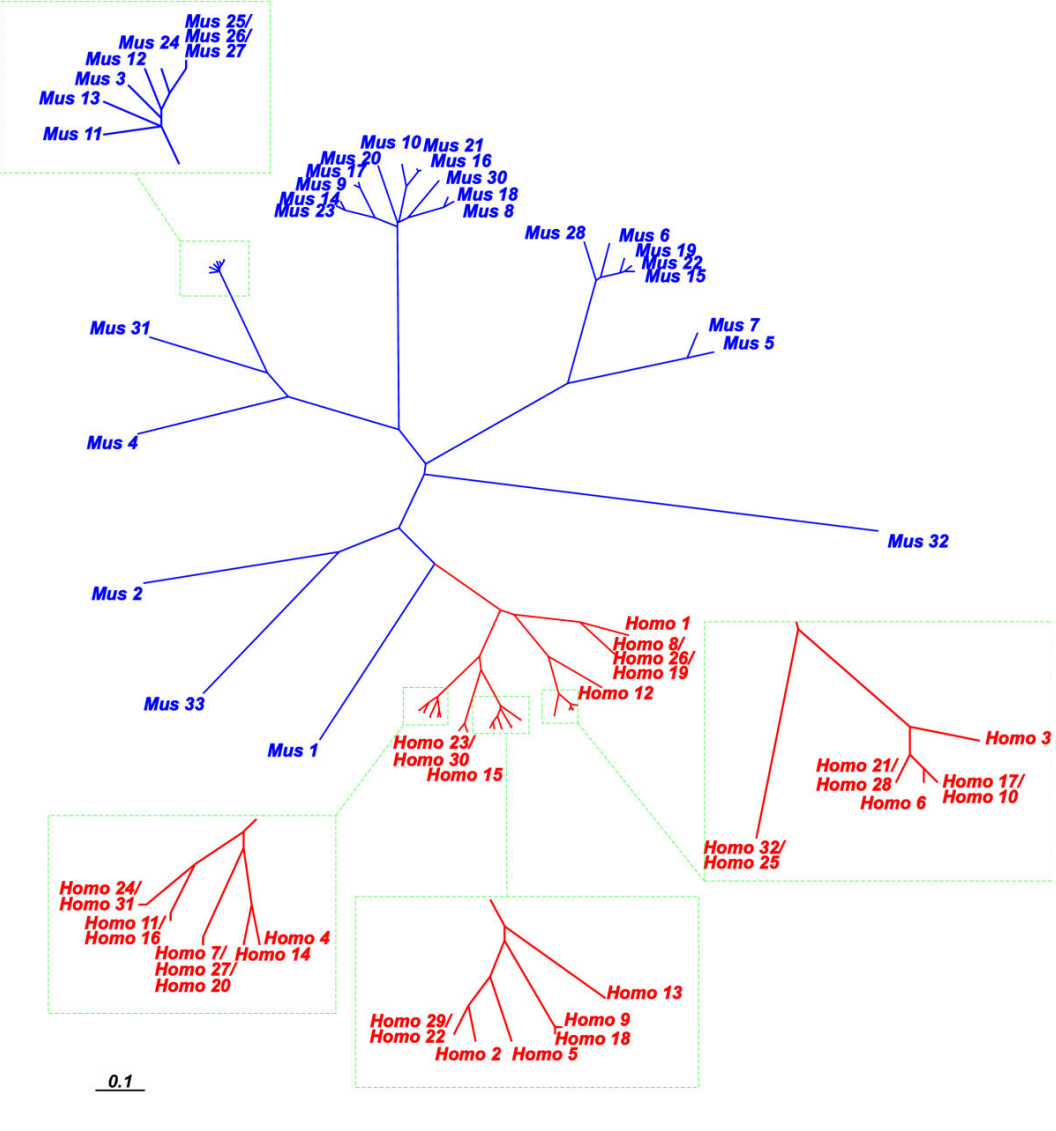
**Phylogenetic relationships of mouse and human full-length *PRAME *homologues, inferred using *K*_*S *_as a distance metric. **Mouse *PRAME *homologues (blue lineages) are monophyletic, as are human *PRAME *homologues (red lineages).

A phylogenetic tree constructed from human and mouse *PRAME *gene *K*_*S *_values revealed that mouse sequences are monophyletic, as are human sequences (Figure 2). No pair of mouse and human *PRAME *genes thus possesses a simple 1:1 orthology relationship. This is a striking result since the vast majority (approximately 80%) of mouse genes possess a single human ortholog [29]. As predicted earlier [11], many human *PRAME *genes thus have arisen by duplication recently in the primate lineage. What was unexpected, however, is that all mouse, and similarly all human, *PRAME *sequences have arisen by duplication events that occurred since their last common ancestor, approximately 85 MYA.

### Human *PRAME *genes have frequently and recently duplicated

**Figure 3:**
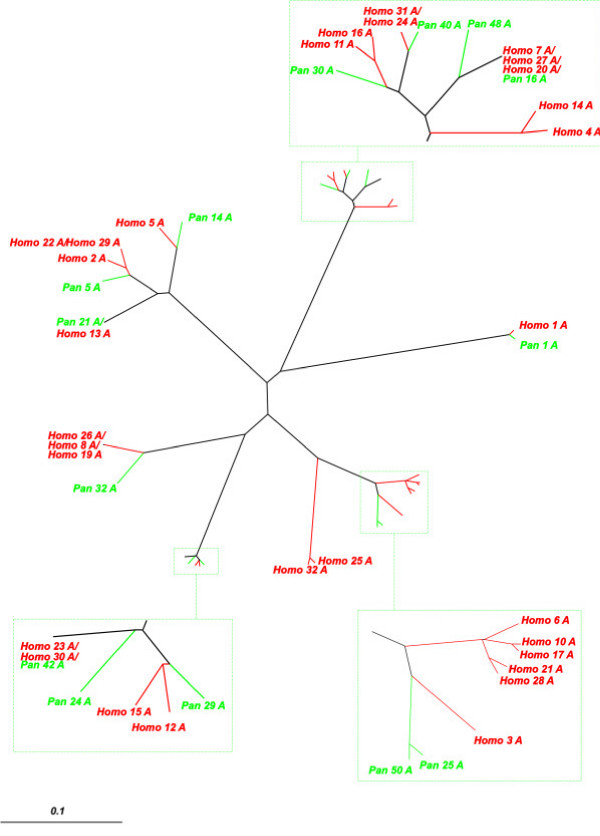
**Phylogenetic relationships of exons A of human and chimpanzee *PRAME *homologues, inferred using *K*_*S *_as a distance metric. **Phylogenetic relationships derived using alignments of exons B and C are available as Additional files 1 and 2. *Homo_9 *and *Homo_18 *are not shown, as these pseudogenes each appears to lack exon A.

Three further phylogenetic trees compared human and chimpanzee *K*_*S *_values from alignments of each of the three *PRAME *exons (Figure 3; Additional Information). Each of these trees indicates that *PRAME *gene sequence duplicated frequently in the terminal human branch (i.e. the lineage from the common ancestor of humans and chimpanzees to humans).

**Figure 4:**
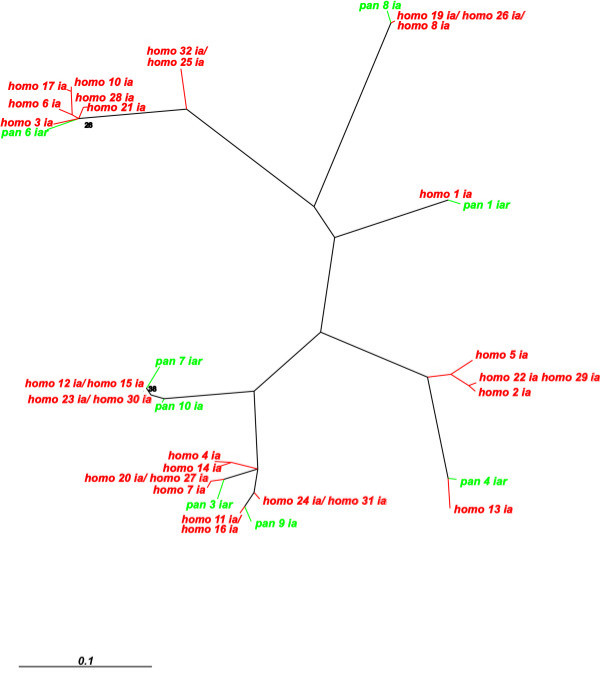
**Phylogenetic relationships of introns *a *of human and chimpanzee *PRAME *homologues, inferred using *K*>_*I *_as a distance metric, and a neighbour-joining tree. **Percentage bootstrap support (1000 iterations) is shown on branches where the support was less than 50%. Phylogenetic relationships derived using an alignment of intron *b *is available as Additional file 3.

**Figure 5:**
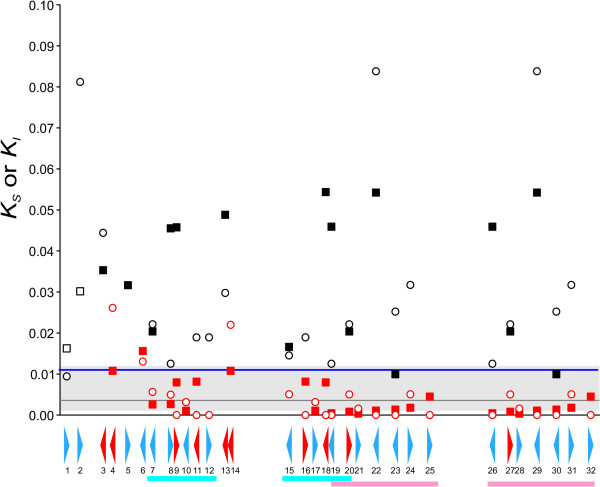
**Scatter plot of the lowest neutral rate estimates (either *K*_*S *_calculated from exon, or *K*_*I *_for intron, alignments) for human *PRAME *genes and either their human paralogues (indicated in red) or their chimpanzee orthologues (indicated in black). **Circles represent averages of intronic rate (*K*_*I*_) estimates, whereas squares represent averages of exonic rate (*K*_*S*_) estimates. The horizontal axis represents genomic location within a 0.74 Mb region of human chromosome 1 (see Figure 1). Two recent segmental duplications (*Homo_7–12 *and *15–20*, and *Homo_19–25 *and *26–32*; see text) are highlighted in blue and pink, respectively. The dark line represents the median *K*_*S *_value (3.58 × 10^-3^) for human paralogues. The grey band identifies 25–75% of this median value (second and third quartiles). The blue line represents the median *K*_*S *_(0.011) for human-chimpanzee coding sequence [30, 31]. The exonic *K*_*S *_value for *Homo_12 *vs *Homo_15 *is not shown due to incongruencies in *K*_*S*_-derived phylogenetic trees (see text). *Homo*-*Pan *rate estimates are missing when the most-closely related sequences, that are available, are relatively divergent *K*_*I *_or *K*_*S *_> 0.1. These missing values are likely to reflect the incompleteness of the current chimpanzee genome assembly. *Homo*-*Homo *rate estimates are missing for 4 genes (*Homo_1*, *3*, *5 *and *13*) which appear not to have duplicated recently (*K*_*I *_or *K*_*S *_> 0.1).

Importantly, many pairs of human *PRAME *genes, and their constituent exons (Figure 3) and introns (Figure 4), were found to exhibit low synonymous rates (Figure 5) that are more typical of duplications in the terminal human branch, than they are of duplications that occurred prior to the common ancestor of humans and chimpanzees, approximately 6–7 MYA [1]. Later in the manuscript we return to the issue of whether these recently-duplicated human genes are present or absent from the chimpanzee genome.

### Assignment of human paralogues and chimpanzee orthologues

By testing for congruency among the three exon (*K*_*S*_) and the two intron (*K*_*I*_) trees (Figures 3 and 4; Additional Information Files 1, 2, 3) we were able to identify the closest human paralogue to each human *PRAME *gene. The set of assignments was found to be unambiguous and internally consistent, with one notable exception: *Homo_15 *and *Homo_12 *are almost identical in their first two exons but are divergent in their exons C. Upon closer inspection it appears that either genome assembly error has generated a chimaeric *Homo_12 *gene, or else its exon C and a portion of intron *b*, have been subjected to inter-locus gene conversion with an, as yet unknown, *PRAME *homologue. Consequently, in subsequent evolutionary rate calculations, comparisons between exons C from *Homo_12 *and *Homo_15 *have been discarded. All human *PRAME *genes, with only 4 exceptions (*Homo_1, Homo_3, Homo_5 *and *Homo_13*), are little diverged (*K*_*S *_< 0.1 or *K*_*I *_< 0.1; Figure 5) from another human gene, and are thus part of a pair of sequence similar paralogues which have apparently been generated by a recent gene duplication.

A similar protocol was adopted to identify chimpanzee orthologues of human *PRAME *exons and introns. For each human exon (or intron), we assigned as its orthologue the chimpanzee exon (or intron) with the lowest *K*_*S *_(or *K*_*I*_) value from the tree, whilst checking to see that these values were approximately 0.011, the median *K*_*S *_value between chimpanzee and human orthologues [30,31]. This process resulted in at least one orthology assignment to the exons or introns of all but 9 (*Homo_4, Homo_6, Homo_10, Homo_14, Homo_17, Homo_21, Homo_25, Homo_28*, and *Homo_32*) of the human *PRAME *homologues; these missing orthologues can be assumed to be present in the chimpanzee genome but absent from its current assembly. For each human orthologue, we then examined the chimpanzee genome assembly for contiguity of its assigned chimpanzee orthologous exons and introns. For example, *Pan_1_A, Pan_2_B *and *Pan_3_C*, which are the chimpanzee orthologues of the three exons of *Homo_1*, appear consecutively within the chimpanzee genome sequence, complete with intervening intronic sequence, and thus were assigned as a full length chimpanzee *PRAME*, *Pan_1*. Several chimpanzee orthologue exon pairs appeared not to be contiguous in the current assembly, which again indicates that considerable additional data and attention will be required to provide an accurate assembly of this region.

### Pseudogenes

Of 32 HSA1 *PRAME *homologues, 10 are predicted to be pseudogenes. A similar proportion of chimpanzee *PRAME *exons are disrupted by at least one stop codon: 19 (3 exon A, 7 exon B and 9 exon C) out of 59 chimpanzee predicted exons contain at least one such disruption. It is probable that some of these are due merely to sequencing or assembly errors due to the low (4-fold) statistical coverage of the chimpanzee genome.

We can safely infer that at least three of these pseudogenes (*Homo_9, Homo_13, and Homo_18*) were present in the common ancestor to both human and chimpanzee simply because in each case the disruptions coincide between orthologues. Five human sequences (*Homo_3*, *Homo_11*, *Homo_16*, *Homo_20 *and *Homo_27*) appear to have become pseudogenes in the hominin lineage as a result of disruptions which are absent from their chimpanzee orthologues. *Homo_20 *and *Homo_27*, which differ by only two synonymous substitutions, acquired their disrupting mutation (a stop codon) only recently, since their divergence from the *Homo_7 *gene, within the last 1 MY (see below).

### Dating segmental duplications in the human genome

The branching order of human genes, both from exon *K*_*S*_-based trees (Figure 3; Additional Information) and from intron *K*_*I*_-based trees (Figure 4; Additional Information), indicates that two large-scale duplication events occurred recently in a human ancestral genome. The most recent event appears to have been a single tandem duplication of 7 *PRAME *homologues to generate a pair of segments encompassing genes *Homo_19–25 *and genes *Homo_26–32 *(Figure 1; Figure 5).

We can estimate the age of this duplication using the neutral rate estimates as a molecular clock and calibrating this by the divergence time (6–7 MY) between the human and chimpanzee lineages (see Figure 3 and Additional Information). Previous large-scale studies have shown that the median *K*_*S *_value between human and chimpanzee orthologues is 0.011 [30,31]. The mean *K*_*S *_value between the seven *Homo_19–32 *genes and their assigned orthologues in chimpanzee was found to be 0.00995. Divergence between these regions of HSA1 and PTR1 thus is typical of these genomes as a whole.

We expect, therefore, that pairs of human paralogues possessing *K*_*S *_values less than approximately 0.01 are likely to have arisen in the terminal human branch, within the past 6–7 MY, whereas human paralogues possessing *K*_*S *_values greater than 0.01 arose due to duplications that occurred prior to the divergence of chimpanzee and human lineages. We calculated that the seven least divergent pairs between *Homo_19–25 *and *Homo_26–32 *exhibit a mean divergence of 1.46 × 10^-3 ^which is nearly seven-fold lower than the chimpanzee-human divergence. This indicates an age for this duplication of approximately (1.46 × 10^-3 ^/9.95 × 10^-3^) × 6 ≈ 0.9 MY. A similar calculation using intronic nucleotide substitution rates *K*_*I *_predicts an age of 0.8 MY. As these predicted ages considerably postdate the split between chimpanzee and human lineages (6–7 MYA), the tandem duplication of *Homo_19–25 *and *Homo_26–32 *genes appears to have been a hominin-specific event.

This conclusion is reinforced by the high identity of genomic sequence between the two duplications. Genomic sequences encompassing *Homo_19–25 *(HSA1 bases 13132294–13272173) and *Homo_26–32 *(bases 13353079–13493033) *PRAME *genes are 99.82% identical (161 mismatched bases over ~140 kb). 0.18% divergence, again, is almost seven-fold lower than 1.23%, the average divergence between human and chimpanzee sequence [8,31,32]. This divergence is also twice the average polymorphism rate (0.08%;  [11,33,34]) between human individuals and in the human genome assembly. This most recent large-scale duplication of human *PRAME *genes thus appears to be recent, with respect to the human-chimpanzee divergence event, but ancient, compared with the appearance of most human polymorphisms, within approximately the last 0.10 MY [35].

A more ancient, but still apparently hominin-specific, large, segmental and inverted duplication is that of *Homo_7–12 *and *Homo_15–20 PRAME *genes (Figure 1; Figure 5). This duplication's average divergence (mean *K*_*I *_= 0.00275; mean *K*_*S *_= 0.00447) is 2.2–3.6-fold smaller than that expected divergence (≈ 0.010, see above) for human-chimpanzee comparisons, which corresponds to an estimated divergence time of between 1.7 and 2.7 MYA. These estimates again considerably postdate the chimpanzee-human divergence.

### Copy number polymorphisms (CNPs)

The recent segmental duplications of human *PRAME *genes suggest that this region of HSA1 might contain CNPs within the human population. By querying the database of genomic variants [36] we determined that HSA1p36.21, which encompasses these *PRAME *genes, is one of only 11 polymorphic loci found in two large-scale CNP investigations [12,36,37] (see Figure 1). This implies that not only has this region undergone two large-scale duplications in ~ 3 MY, but that there have been additional, more recent, duplications which are not fixed in the human population and have not been captured in the human genome reference sequence.

### Positive selection of *PRAME *genes

Gene duplication in a genome provides a substrate upon which selection may act. The preservation of duplicates without disruption to their open-reading frames over millennia is itself an indication that these duplicates confer a selective benefit to the host organism. More direct evidence of positive selection comes from the elevated values of the ratio of *K*_*A*_, the number of nonsynonymous (amino acid changing) substitutions per nonsynonymous site, to *K*_*S*_. After discarding closely-related sequences (*K*_*S *_< 0.02), the median *K*_*A*_/*K*_*S *_ratio between pairs of human *PRAME *genes is 0.73, and 19 gene pairs exhibit *K*_*A*_/*K*_*S *_ratios greater than 1, with a maximum value of 1.73 between *Homo_6 *and *Homo_10*. These values are considerably higher than the average ratio between human and rodent single gene orthologues (median *K*_*A*_/*K*_*S*_~ 0.12) [29].

*K*_*A*_/*K*_*S *_values of approximately 1 might be due to positive selection of nonsynonymous nucleotide substitutions, or to reduced selective constraints due to the loss of *PRAME *genes' functions. In order to distinguish between these hypotheses, and to further investigate the evolution of these genes, we used codeml [38-40] to infer positive selection at single sites within multiple alignments of human or mouse *PRAME *genes.

**Figure 6:**
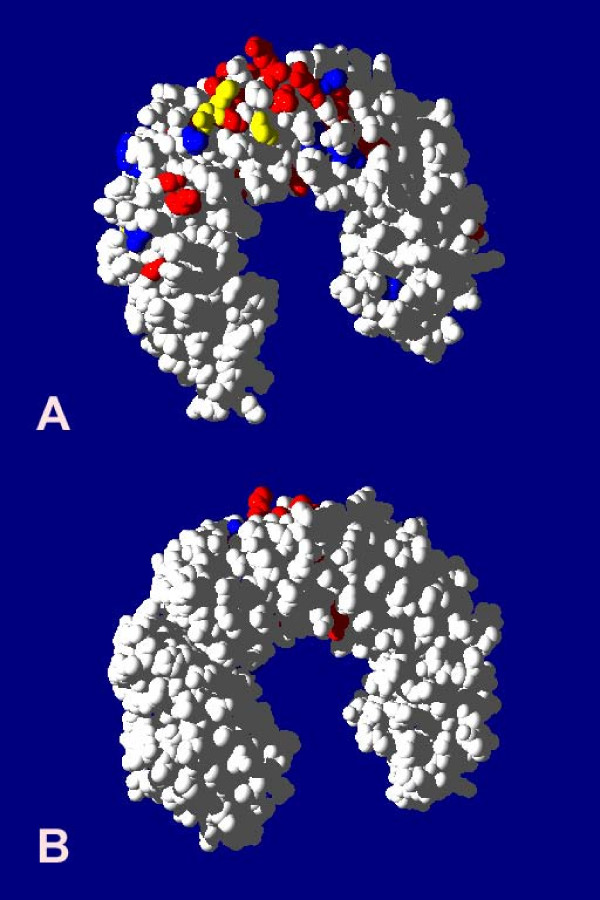
Structure of porcine ribonuclease inhibitor (PDB code 2BNH) with amino acid sites that are positively-selected among human and mouse PRAME proteins shown in red and blue, respectively, ((A) front view, (B) rear view).

Among human *PRAME *genes, a large number (30) of amino acid sites were identified as having been subject to positive selection. By mapping these sites to a homologous protein structure, that of porcine ribonuclease inhibitor, we observed that these sites aggregate to form a pronounced cluster on one exterior face (Figure 6). The majority of these sites would thus be available to participate in binding interactions. A similar analysis of mouse *PRAME *genes also demonstrated the impact of positive selection: 17 positively selected sites were identified, of which 4 coincide with such sites among human *PRAME *genes.

## Discussion

Our findings demonstrate an extraordinarily rapid expansion within this *PRAME *gene family that occurred independently in both primate and rodent lineages. Given the high conservation of gene order among chicken, dog, human and rodent genomes, we can date the origin of this cluster to between approximately 95 and 85 MYA [26]. This is because *PRAME *homologues are undetectable in the orthologous region of the chicken and dog genomes, but are present in syntenic portions of primate and rodent genomes. Thereafter, many episodes of gene duplication have occurred in both primate and rodent lineages.

In order to infer the most recent of these duplication events, we identified 13 pairs of human *PRAME *paralogues which appear to have arisen by duplication since the common ancestor with chimpanzees: their divergence is considerably less than both the expected and the observed divergence between orthologous human and chimpanzee sequence (Figure 1 and Figure 5). Using a molecular clock, and a palaeontological calibration of divergence between these two species of 6–7 MYA [1], we estimate that two large segmental duplications of *PRAME *genes occurred independently in the terminal human branch, within approximately the past 3 MY.

The low divergence of these human paralogues, compared with the divergence between chimpanzee and human orthologues, argues strongly that chimpanzee lacks single orthologues of many, if not all, of these human duplicated genes. Insufficient nucleotide substitutions at synonymous and intronic sites have accumulated to indicate that *Homo_7–12 *and *Homo_15–32 *genes were all present in single copies in the common ancestor of chimpanzee and human. Even when the chimpanzee genome is completed, we thus expect that chimpanzee single orthologues of these genes will not be identified.

In addition to these duplications which are apparent in the human genome assembly, it appears, from two independent studies [36,12], that the region of human chromosome 1 (HSA1) containing these *PRAME *genes is copy number polymorphic. These human genes are present in different numbers among the human population, thus providing further evidence that human-specific duplications are a feature of this region. CNPs are thought not to be selectively neutral [12]. Their persistence in the human population suggests, rather, that at least a subset of CNPs may be adaptive.

In support of this hypothesis, we found that a large number (30) of codons in the HSA1 *PRAME *family have been subject to positive selection. 26 of these adaptive codons were confirmed using the "sitewise likelihood-ratio" (SLR) method [41] (data not shown). These sites are clustered onto one surface in a homology model of protein structure, thereby demarcating a likely surface-accessible functional site. Mouse *PRAME *genes also contain a large number (17) of positively selected sites, which cluster within a site equivalent to that for human *PRAME *genes (Figure 6).

Expansion of this *PRAME *gene family has occurred independently in both primate and rodent lineages. In each of these lineages, *PRAME *genes appear to have evolved by 'birth-and-death' processes, such as occurs for immunity genes [42]: genes both persist as duplications, and are lost by pseudogene creation. Sequence similarities between paralogues, however, could have arisen also from concerted evolution, as the result of homologous recombination, in particular, gene conversion and unequal crossing over [43]. Nevertheless, the recent origin of the *PRAME *progenitor gene just prior to the common ancestor of primates and rodents, and its rapid duplication thereafter, and the occurrence of CNPs in the human population, each indicates that the predominant process in this expansion has been gene duplication. Moreover, the congruency of dendrograms associated with separate exons or introns (Figures 3 and 4, and Additional files 1, 2, 3), and the tandem segmental duplications we have inferred (Figure 1; Figure 5), also argue against concerted evolution as a dominant evolutionary mechanism.

*PRAME *genes have arisen by rapid gene duplication and pseudogene creation, and their sequences have been subject to positive selection. Nevertheless, the adaptive advantages conferred on these genes by their duplication and sequence diversification remain unclear, as are the genes' functions in normal tissues. Their expression profile often is limited to testes and to a wide variety of tumors, which suggests that PRAME proteins might perform important mitotic roles in rapidly dividing cells. This is consistent with the observation that a *PRAME*-like gene (*oogenesin*) in mouse accumulates in the nucleus only at the late one-cell and early two-cell stages of early embryos [22].

At least one of the mouse orthologues of these *PRAME *genes is expressed in spermatogonial cells [21]. Interestingly, it is known that a mutation in *FGFR2 *expressed in these cells confers a selective advantage, thereby leading to clonal expansion similar to that seen in tumours [44]. We suggest that similar nonsynonymous substitutions in *PRAME *genes might have conferred comparable benefits to these cells, and these have driven positive selection for both gene duplication and sequence change.

The evidence thus points mainly to darwinian selection in spermatogonia, and adaptive evolution of *PRAME *genes thus may be thought not to have phenotypic consequences at larger anatomical scales. Nevertheless, a gene with a similar function, and a similar evolutionary history, to *PRAME *genes, has been proposed to having contributed to anatomical adaptations during recent hominid evolution. The *Abnormal spindle-like microcephaly associated (ASPM) *gene, which has evolved rapidly in the great apes [45], has roles in both spermatogenesis and oogenesis in *Drosophila *[46,47]. When mutated in humans, this results in primary microcephaly, which is manifested by a greatly reduced brain size [6]. Further examination of human *PRAME *genes' functions should assist in our understanding of the cellular and physiological consequences of its recent and rapid evolution.

## Conclusion

Whatever the selective advantages conferred by the *PRAME *genes discussed here, it is apparent from their recent introduction to an ancestral chromosome of primates and rodents that they benefited only these lineages. Moreover, the HSA1 *PRAME *gene family has expanded further, in particular by two large segmental duplications in the past 3 MY and further duplications that manifest themselves as copy number polymorphisms in the human population. These extremely rapid duplications, taken together with strong evidence for darwinian adaptation at approximately 30 sites among human *PRAME *genes, indicates that this family has experienced sustained episodes of positive selection during recent hominin history.

## Methods

### Survey of human CT-Antigen genes

We recently identified 41 human gene families that have experienced multiple gene duplications since the divergence of rodent and primate lineages [11]. Among these were 6 families, SSX-like, MAGE, GAGE, XAGE, SAGE and SPAN-X, all encoded on the X chromosome, which exhibit the tissue expression profiles of CT-antigens (CTAs). A seventh CTA family, recently duplicated in our lineage, encode *PRAME *genes that are located on *Homo sapiens *chromosome 1 (HSA1). In an initial survey, only 2 of these 7 families yielded evidence of positive selection at individual sites using codeml (data not shown), using methods and criteria described below. Subsequently, we chose to perform more comprehensive analyses of the *PRAME *gene family because of its larger size (13 human ENSEMBL predicted genes) and its high number of positively selected sites identified.

### Prediction of human *PRAME *genes and exons

The amino acid sequences of 5 *PRAME *homologues were aligned using CLUSTALW [48]. These are genes that were identified by Ensembl [11,49] and lie in a cluster between bases 12550000 and 13100000 of human chromosome 1 (assembly NCBI35). (Many of the remaining 8 ENSEMBL *PRAME *genes were mispredicted.) From this multiple alignment a hidden Markov model (HMM) was constructed [50]. On the basis of strong conservation of a translation initiating methionine codon, presumed non-coding sequence upstream of this codon was discarded.

In order to ensure gene prediction fidelity and completeness, we repredicted *PRAME *homologous genes and pseudogenes from this region of HSA1 (bases 12550000 and 13100000, which include the flanking non-homologous RefSeq genes, *DHRS3 *and *T1A-2*). Gene prediction employed Genewise [51], the PRAME HMM and default parameters. Upon building phylogenetic trees, the predicted *PRAME *homologues were found to be monophyletic, and are only distantly-related to other *PRAME *homologous genes located elsewhere on HSA1 and on HSA22 (data not shown). Pseudogenes were distinguished from genes on the basis of premature stop codons or frameshifts; pseudogenes that are functionally disrupted due only to mutations that occur outside of coding sequence are thus misassigned.

In an independent approach, we also predicted homologues of each of the three protein-coding exons of these *PRAME *genes within this region of HSA1 using HMMs of multiply aligned nucleotide exonic sequence. This procedure resulted in no predictions that were additional to those found using Genewise and full-length gene sequence.

### Prediction of mouse *PRAME *genes

Mouse *PRAME *genes were predicted as described above for human genes, except for the use of known mouse '*PRAME*-like' (*PRAMEL*) genes to derive the HMM used in the Genewise step. Initially a CLUSTALW alignment of three known mouse RefSeq genes (*PRAMEL1 *[RefSeq code: NM_031377.1], *PRAMEL3 *[NM_031390.1] and *PRAMEL4 *[NM_178248.2]) was used for the HMM query template. The 3' ends of the *PRAMEL *genes, however, were found to be relatively divergent. The alignment was thus trimmed back to exclude the ends of the third, and final, exons. Thus the alignment of mouse PRAME proteins is 58 amino acids shorter than that of human PRAME proteins. Mouse genes were predicted within the orthologous region of the mouse genome (*Mus musculus *chromosome 4 [MMU4]; May 2004 assembly; bases 141,850,000–142,800,000) between mouse *DHRS3 *and *T1A-2*. 29 mouse *PRAMEL *genes and pseudogenes were predicted from this approach. An HMM was then derived from a multiple alignment of these sequences and used to query this region of MMU4 in a second round of searches. Four additional predictions were found.

### Prediction of chimpanzee *PRAME *genes and exons

Chimpanzee (*Pan troglodytes) PRAME *genes were predicted as for human genes, as described above, using the HMM derived from the alignment of human PRAME amino acid sequences as query to search a region lying between orthologous *DHRS3 *and *T1A-2 *genes on chimpanzee chromosome 1 (PTR1, bases 10240000–13450000, Nov 2003 assembly). This method identified 17 full-length chimpanzee *PRAME *genes. This gene count was substantially fewer than for the human genome assembly, and may be a consequence of the low (four-fold) statistical coverage of the chimpanzee genome assembly. We reasoned, therefore, that additional non-full-length *PRAME *gene exons might be represented in the chimpanzee genome assembly. We thus predicted homologues of each of the three *PRAME *gene exons in this region using the protocol (described above) that was used for predicting human *PRAME *gene exons.

### Prediction of introns

Human and chimp intron sequences were identified as the sequence intervening between adjacent exons. *PRAME *genes contain 3 coding exons (labelled A, B, C) and 2 intervening introns, labelled intron *a *and intron *b*. For human sequence, these introns were all complete, without gaps, but for chimpanzee sequence only 9 intact intron *a *and 5 intron *b *chimpanzee introns could be identified.

Exon, intron and splice site predictions from these three species were all consistent with the gene structures apparent from available cDNAs, in particular 14 cDNAs mapped to human HSA1 bases 12769277–1349033, 29 cDNAs mapped to mouse MMU4 bases 141852757–142731257, and cDNAs from the eponymous *PRAME *gene on HSA22 (bases 21,215,046–21,218,065). Conservation of gene structures between mammals as diverse as human and mouse, together with conservation of splice sites, indicates that chimpanzee *PRAME *genes also possess an identical gene structure.

### Sequence alignments

Conceptual translations of *PRAME *genes and pseudogenes were aligned using CLUSTAL W [48] and then modified to minimise gaps (see Additional files 1, 2, 3). Stop characters were replaced by 'X'. Estimates of *K*_*A *_and *K*_*S *_for sequence pairs (see below) were calculated from cDNA sequences aligned according to these amino acid multiple alignments.

Human and chimpanzee nucleotide intronic sequences were aligned using DIALIGN-2 [52].

### Exonic evolutionary rates

Codeml [23] was used to conduct site-specific *K*_*A*_*/K*_*S *_analysis on the human and mouse full length *PRAME *predictions. An amino acid alignment and corresponding cDNA alignment were prepared for each analysis. Identified pseudogenes were removed from the alignment because they are likely to be no longer subject to selective constraints.

The maximum likelihood approach of Yang [40] was used to predict sites in a group of cDNA sequences that have been subject to positive selection. Pairs of models were compared by calculating log likelihood values (*l*), which were then compared for significant differences using a Likelihood Ratio Test. The first of each pair of models compared is a simple model where sites are predicted to be associated with *K*_*A*_*/K*_*S *_ratios between 0 and 1. The second is a more complex model that allows adaptive sites: for these, ratios can be greater than 1. If the complex model indicates an estimated *K*_*A*_*/K*_*S *_ratio that is greater than one, and the test statistic (2Δ*l*) is greater than critical values of the Chi square (χ^2^) distribution with the appropriate degree of freedom [53], then positive selection can be inferred. Bayesian probabilities are used to predict which codons in the original data have most likely been subjected to positive selection.

The pairs of simple and complex models we used were: M0 (one-ratio) [54] versus M3 (discrete) [23]; M1 (neutral) versus M2 (selection) [55]; and M7 (beta) versus M8 (beta + ω) [23,40]. Only non-conserved alignment positions predicted to be under positive selection with a posterior probability > 0.90 by all three codeml models were mapped onto a homologous protein structure (Figure 6).

### Intronic evolutionary rates

Using the DIALIGN-2 alignment of intronic sequences, we calculated their genetic distances using the TN93 nucleotide substitution model [56]. We then derived a phylogenetic tree based on the distance matrix using neighbour-joining methods (1000 bootstrap iterations). Numbers of nucleotide substitutions per intronic site between sequence pairs (*K*_*I*_) were then estimated using BASEML [38,39], a maximum likelihood method, and the TN93 nucleotide substitution model. This analysis was implemented using the DAMBE package [57].

### Structure

In order to gain insight into the functional relevance of positively selected sites, we searched the protein sequences of known tertiary structure using PSI-BLAST [25] using a human PRAME sequence (Homo_7; UniProt: YA03_HUMAN) at NCBI using default parameters. Significant sequence similarity (*E *= 1 × 10^-12^) was found after three search iterations to porcine ribonuclease inhibitor, RNI (PDB code 2BNH). Two types of alignment guided the assignment of leucine-rich repeats (LRRs) to human PRAME sequences. First, the BLAST alignment, and second the optimal and suboptimal alignments of PRAME sequence against the SMART [58] LRR HMM. RNI was first aligned to Homo_7 using these methods, and adjusted manually, and then aligned to the full alignment of all human PRAMEs guided by the Homo_7 alignment. This allowed human PRAME positively selected sites (as identified by the method above) to be mapped to RNI residues. This procedure was also followed to align full-length mouse PRAMEL sequences against RNI. Protein tertiary structure was viewed, manipulated and annotated in Swiss Pdbviewer [59].

### Evolutionary relationships

Phylogenetic relationships were deduced from dendrograms constructed from three types of neutral rate estimates: either (i) *K*_*S *_values of pairwise alignments of full-length coding sequences; (ii) *K*_*S *_values of pairwise alignments of coding sequences from exons A, B or C; or (iii) *K*_*I *_values of pairwise alignments of intronic sequences from introns *a *or *b*. Dendrograms were constructed using code based on PHYLIP [60] which uses the Fitch-Margoliash criteria to build trees with contemporaneous tips. The dendrograms were visualised in njplot and treeview [61].

### Copy number polymorphisms (CNPs)

The database of Genomic variants ([62,12,36]) was queried to determine whether the human *PRAME *region has been determined previously to harbour CNPs.

## List of abbreviations

BLAST – Basic Local Alignment Search Tool

CNP – copy number polymorphism

CTA – cancer testis antigen

HMM – hidden Markov model

HSA1 – human chromosome 1

MYA – million years ago

NCBI – The National Centre for Biotechnology Information, USA

PDB – protein data bank

PRAME – preferentially expressed antigen of melanoma

PSI-BLAST – Position specific iterative BLAST

SNP – single nucleotide polymorphism

## Authors' contributions

ZB predicted and aligned *PRAME *genes, identified the positively selected sites and drew the trees. ZB also wrote the first draft of the manuscript. LG retrieved the initial gene sequences and developed an analytical pipeline which proved critical to this project. CP participated in sequence alignment, analysis of the data and writing the manuscript, in particular the discussion. All Authors read and approved the final manuscript.

## Supplementary Material

Additional File 1 Phylogenetic relationships of exons B of human and chimpanzee *PRAME *homologues, inferred using *K*_*S *_as a distance metric Click here for file

Additional File 2 Phylogenetic relationships of exons C of human and chimpanzee *PRAME *homologues, inferred using *K*_*S*_as a distance metric Click here for file

Additional File 3 **Phylogenetic relationships of introns *b *of human and chimpanzee *PRAME *homologues, inferred using *K*_*I *_as a distance metric, and a neighbour-joining tree. **Percentage bootstrap support (1000 iterations) is shown on branches where the support was less than 50%. In the case of *Homo_18_ib *and *Homo_9_ib*, the branches leading to these sequences were too small to display the bootstrap values clearly. The bootstrap support values for these branches are 25% and 45% respectively. Click here for file

Additional File 4 **Human_prames_pep.aln **• Clustal format • Human PRAME predictions • This contains an alignment of human predicted PRAME polypeptides. This alignment (without pseudogenes) was used to identify positively-selected sites. Click here for file

Additional File 5 **Human_prames_cdna.fa **• FASTA format • Human PRAME cdnas • This contains the human PRAME cDNAs, as predicted by Genewise. Click here for file

Additional File 6 **Human_mouse_2BNH.aln **• Clustal format • Human and mouse PRAME alignment with positively selected sites. • This shows all human and mouse PRAMEs (including pseudogenes) aligned. Two lines, homo_sites and mus_sites, indicate positively selected sites ('X') in the human PRAMEs and mouse PRAMEs respectively. This alignment (without pseudogenes) was used to identify positively-selected sites. Click here for file

Additional File 7 **Human_chimp_exonA_cdna.fa **• FASTA format • Human and chimpanzee exon A cDNA • This contains the human and chimp exon A cDNA as predicted by Genewise. Click here for file

Additional File 8 **Human_chimp_exonB_cdna.fa **• FASTA format • Human and chimpanzee exon B cDNA • This contains the human and chimp exon B cDNA as predicted by Genewise. Click here for file

Additional File 9 **Human_chimp_exonC_cdna.fa **• FASTA format • Human and chimpanzee exon C cDNA • This contains the human and chimp exon C cDNA as predicted by Genewise. Click here for file

Additional File 10 **Human_chimp_exonA_pep.aln **• Clustal format • Human and chimpanzee exon A peptide alignment • This contains human and chimp exons A peptide sequences aligned. This alignment was used to calculate the *K*_*S *_distances between sequences. Click here for file

Additional File 11 **Human_chimp_exonB_pep.aln **• Clustal format • Human and chimpanzee exon B peptide alignment • This contains human and chimp exons B peptide sequences aligned. This alignment was used to calculate the *K*_*S *_distances between sequences. Click here for file

Additional File 12 **Human_chimp_exonC_pep.aln **• Clustal format • Human and chimpanzee exon C peptide alignment • This contains human and chimp exons C peptide sequences aligned. This alignment was used to calculate the *K*_*S *_distances between sequences. Click here for file

Additional File 13 **Chimp_exonA_cdna.fa **• FASTA format • Chimpanzee exon A cDNA • This contains the (unaligned) chimp exons A cDNA as predicted by Genewise. Click here for file

Additional File 14 **Chimp_exonB_cdna.fa **• FASTA format • Chimpanzee exon B cDNA • This contains the (unaligned) chimp exons B cDNA as predicted by Genewise. Click here for file

Additional File 15 **Chimp_exonC_cdna.fa **• FASTA format • Chimpanzee exon C cDNA • This contains the (unaligned) chimp exons C cDNA as predicted by Genewise. Click here for file

Additional File 16 **Human_prames_info.xls **• Excel spreadsheet format • Human PRAME information • This excel spread sheet contains details of human PRAMEs, including translational start and end positions, length, chimpanzee orthologues and pseudogene assignment. Click here for file

Additional File 17 **chimp_prames_info.xls **• Excel spreadsheet format • Full length chimpanzee PRAME information • This excel spread sheet contains details of full length chimpanzee PRAMEs. It indicates exons which constitute each PRAME and contains details of pseudogene assignment. Click here for file

Additional File 18 **Human_chimp_introna.aln **• Clustal format • Human and chimpanzee introns a sequence • This contains human and chimpanzee introns a sequence aligned. Click here for file

Additional File 19 **Human_chimp_intronb.aln **• Clustal format • Human and chimpanzee introns b sequence • This contains human and chimpanzee intron b sequences aligned. Click here for file

## References

[B1] Brunet M, Guy F, Pilbeam D, Mackaye HT, Likius A, Ahounta D, Beauvilain A, Blondel C, Bocherens H, Boisserie JR, De Bonis L, Coppens Y, Dejax J, Denys C, Duringer P, Eisenmann V, Fanone G, Fronty P, Geraads D, Lehmann T, Lihoreau F, Louchart A, Mahamat A, Merceron G, Mouchelin G, Otero O, Pelaez Campomanes P, Ponce De Leon M, Rage JC, Sapanet M, Schuster M, Sudre J, Tassy P, Valentin X, Vignaud P, Viriot L, Zazzo A, Zollikofer C (2002). A new hominid from the Upper Miocene of Chad, Central Africa. Nature.

[B2] Ruvolo M (2004). Comparative primate genomics: the year of the chimpanzee. Curr Opin Genet Dev.

[B3] Enard W, Przeworski M, Fisher SE, Lai CS, Wiebe V, Kitano T, Monaco AP, Paabo S (2002). Molecular evolution of FOXP2, a gene involved in speech and language. Nature.

[B4] Stedman HH, Kozyak BW, Nelson A, Thesier DM, Su LT, Low DW, Bridges CR, Shrager JB, Minugh-Purvis N, Mitchell MA (2004). Myosin gene mutation correlates with anatomical changes in the human lineage. Nature.

[B5] Winter H, Langbein L, Krawczak M, Cooper DN, Jave-Suarez LF, Rogers MA, Praetzel S, Heidt PJ, Schweizer J (2001). Human type I hair keratin pseudogene phihHaA has functional orthologs in the chimpanzee and gorilla: evidence for recent inactivation of the human gene after the Pan-Homo divergence. Hum Genet.

[B6] Bond J, Roberts E, Mochida GH, Hampshire DJ, Scott S, Askham JM, Springell K, Mahadevan M, Crow YJ, Markham AF, Walsh CA, Woods CG (2002). ASPM is a major determinant of cerebral cortical size. Nat Genet.

[B7] Ponting CP, Jackson A (2005). Evolution of primary microcephaly genes and the enlargement of primate brain. Curr Opin genet dev.

[B8] Ebersberger I, Metzler D, Schwarz C, Paabo S (2002). Genomewide comparison of DNA sequences between humans and chimpanzees. Am J Hum Genet.

[B9] Shi J, Xi H, Wang Y, Zhang C, Jiang Z, Zhang K, Shen Y, Jin L, Yuan W, Lin J, Hua Q, Wang F, Xu S, Ren S, Zhao G, Chen Z, Huang W (2003). Divergence of the genes on human chromosome 21 between human and other hominoids and variation of substitution rates among transcription units. Proc Natl Acad Sci U S A.

[B10] University of Washington in St Louis. Chimpanzee Sequencing project.. http://genomeold.wustl.edu/projects/chimp/contents/talking_points.pdf.

[B11] Consortium IHGS (2004). Finishing the euchromatic sequence of the human genome. Nature.

[B12] Sebat J, Lakshmi B, Troge J, Alexander J, Young J, Lundin P, Maner S, Massa H, Walker M, Chi M, Navin N, Lucito R, Healy J, Hicks J, Ye K, Reiner A, Gilliam TC, Trask B, Patterson N, Zetterberg A, Wigler M (2004). Large-scale copy number polymorphism in the human genome. Science.

[B13] King MC, Wilson AC (1975). Evolution at two levels in humans and chimpanzees. Science.

[B14] Keightley PD, Lercher MJ, Eyre-Walker A (2005). Evidence for widespread degradation of gene control regions in hominid genomes. PLoS Biol.

[B15] Tuzun E, Sharp AJ, Bailey JA, Kaul R, Morrison VA, Pertz LM, Haugen E, Hayden H, Albertson D, Pinkel D, Olson MV, Eichler EE (2005). Fine-scale structural variation of the human genome. Nat Genet.

[B16] Scanlan MJ, Gure AO, Jungbluth AA, Old LJ, Chen YT (2002). Cancer/testis antigens: an expanding family of targets for cancer immunotherapy. Immunol Rev.

[B17] Ikeda H, Lethe B, Lehmann F, van Baren N, Baurain JF, de Smet C, Chambost H, Vitale M, Moretta A, Boon T, Coulie PG (1997). Characterization of an antigen that is recognized on a melanoma showing partial HLA loss by CTL expressing an NK inhibitory receptor. Immunity.

[B18] van Baren N, Chambost H, Ferrant A, Michaux L, Ikeda H, Millard I, Olive D, Boon T, Coulie PG (1998). PRAME, a gene encoding an antigen recognized on a human melanoma by cytolytic T cells, is expressed in acute leukaemia cells. Br J Haematol.

[B19] Emes RD, Goodstadt L, Winter EE, Ponting CP (2003). Comparison of the genomes of human and mouse lays the foundation of genome zoology. Hum Mol Genet.

[B20] Ross MT, Grafham DV, Coffey AJ, Scherer S, McLay K, Muzny D, Platzer M, Howell GR, Burrows C, Bird CP, Frankish A, Lovell FL, Howe KL, Ashurst JL, Fulton RS, Sudbrak R, Wen G, Jones MC, Hurles ME, Andrews TD, Scott CE, Searle S, Ramser J, Whittaker A, Deadman R, Carter NP, Hunt SE, Chen R, Cree A, Gunaratne P, Havlak P, Hodgson A, Metzker ML, Richards S, Scott G, Steffen D, Sodergren E, Wheeler DA, Worley KC, Ainscough R, Ambrose KD, Ansari-Lari MA, Aradhya S, Ashwell RI, Babbage AK, Bagguley CL, Ballabio A, Banerjee R, Barker GE, Barlow KF, Barrett IP, Bates KN, Beare DM, Beasley H, Beasley O, Beck A, Bethel G, Blechschmidt K, Brady N, Bray-Allen S, Bridgeman AM, Brown AJ, Brown MJ, Bonnin D, Bruford EA, Buhay C, Burch P, Burford D, Burgess J, Burrill W, Burton J, Bye JM, Carder C, Carrel L, Chako J, Chapman JC, Chavez D, Chen E, Chen G, Chen Y, Chen Z, Chinault C, Ciccodicola A, Clark SY, Clarke G, Clee CM, Clegg S, Clerc-Blankenburg K, Clifford K, Cobley V, Cole CG, Conquer JS, Corby N, Connor RE, David R, Davies J, Davis C, Davis J, Delgado O, Deshazo D, Dhami P, Ding Y, Dinh H, Dodsworth S, Draper H, Dugan-Rocha S, Dunham A, Dunn M, Durbin KJ, Dutta I, Eades T, Ellwood M, Emery-Cohen A, Errington H, Evans KL, Faulkner L, Francis F, Frankland J, Fraser AE, Galgoczy P, Gilbert J, Gill R, Glockner G, Gregory SG, Gribble S, Griffiths C, Grocock R, Gu Y, Gwilliam R, Hamilton C, Hart EA, Hawes A, Heath PD, Heitmann K, Hennig S, Hernandez J, Hinzmann B, Ho S, Hoffs M, Howden PJ, Huckle EJ, Hume J, Hunt PJ, Hunt AR, Isherwood J, Jacob L, Johnson D, Jones S, de Jong PJ, Joseph SS, Keenan S, Kelly S, Kershaw JK, Khan Z, Kioschis P, Klages S, Knights AJ, Kosiura A, Kovar-Smith C, Laird GK, Langford C, Lawlor S, Leversha M, Lewis L, Liu W, Lloyd C, Lloyd DM, Loulseged H, Loveland JE, Lovell JD, Lozado R, Lu J, Lyne R, Ma J, Maheshwari M, Matthews LH, McDowall J, McLaren S, McMurray A, Meidl P, Meitinger T, Milne S, Miner G, Mistry SL, Morgan M, Morris S, Muller I, Mullikin JC, Nguyen N, Nordsiek G, Nyakatura G, O'Dell CN, Okwuonu G, Palmer S, Pandian R, Parker D, Parrish J, Pasternak S, Patel D, Pearce AV, Pearson DM, Pelan SE, Perez L, Porter KM, Ramsey Y, Reichwald K, Rhodes S, Ridler KA, Schlessinger D, Schueler MG, Sehra HK, Shaw-Smith C, Shen H, Sheridan EM, Shownkeen R, Skuce CD, Smith ML, Sotheran EC, Steingruber HE, Steward CA, Storey R, Swann RM, Swarbreck D, Tabor PE, Taudien S, Taylor T, Teague B, Thomas K, Thorpe A, Timms K, Tracey A, Trevanion S, Tromans AC, d'Urso M, Verduzco D, Villasana D, Waldron L, Wall M, Wang Q, Warren J, Warry GL, Wei X, West A, Whitehead SL, Whiteley MN, Wilkinson JE, Willey DL, Williams G, Williams L, Williamson A, Williamson H, Wilming L, Woodmansey RL, Wray PW, Yen J, Zhang J, Zhou J, Zoghbi H, Zorilla S, Buck D, Reinhardt R, Poustka A, Rosenthal A, Lehrach H, Meindl A, Minx PJ, Hillier LW, Willard HF, Wilson RK, Waterston RH, Rice CM, Vaudin M, Coulson A, Nelson DL, Weinstock G, Sulston JE, Durbin R, Hubbard T, Gibbs RA, Beck S, Rogers J, Bentley DR (2005). The DNA sequence of the human X chromosome. Nature.

[B21] Wang PJ, McCarrey JR, Yang F, Page DC (2001). An abundance of X-linked genes expressed in spermatogonia. Nat Genet.

[B22] Minami N, Aizawa A, Ihara R, Miyamoto M, Ohashi A, Imai H (2003). Oogenesin is a novel mouse protein expressed in oocytes and early cleavage-stage embryos. Biol Reprod.

[B23] Yang Z, Nielsen R (2000). Estimating synonymous and nonsynonymous substitution rates under realistic evolutionary models. Mol Biol Evol.

[B24] Dade S, Callebaut I, Mermillod P, Monget P (2003). Identification of a new expanding family of genes characterized by atypical LRR domains. Localization of a cluster preferentially expressed in oocyte. FEBS Lett.

[B25] Altschul SF, Madden TL, Schaffer AA, Zhang J, Zhang Z, Miller W, Lipman DJ (1997). Gapped BLAST and PSI-BLAST: a new generation of protein database search programs. Nucleic Acids Res.

[B26] Springer MS, Murphy WJ, Eizirik E, O'Brien SJ (2003). Placental mammal diversification and the Cretaceous-Tertiary boundary. Proc Natl Acad Sci U S A.

[B27] Collier S, Tassabehji M, Sinnott P, Strachan T (1993). A de novo pathological point mutation at the 21-hydroxylase locus: implications for gene conversion in the human genome. Nat Genet.

[B28] Urabe K, Kimura A, Harada F, Iwanaga T, Sasazuki T (1990). Gene conversion in steroid 21-hydroxylase genes. Am J Hum Genet.

[B29] Waterston RH, Lindblad-Toh K, Birney E, Rogers J, Abril JF, Agarwal P, Agarwala R, Ainscough R, Alexandersson M, An P, Antonarakis SE, Attwood J, Baertsch R, Bailey J, Barlow K, Beck S, Berry E, Birren B, Bloom T, Bork P, Botcherby M, Bray N, Brent MR, Brown DG, Brown SD, Bult C, Burton J, Butler J, Campbell RD, Carninci P, Cawley S, Chiaromonte F, Chinwalla AT, Church DM, Clamp M, Clee C, Collins FS, Cook LL, Copley RR, Coulson A, Couronne O, Cuff J, Curwen V, Cutts T, Daly M, David R, Davies J, Delehaunty KD, Deri J, Dermitzakis ET, Dewey C, Dickens NJ, Diekhans M, Dodge S, Dubchak I, Dunn DM, Eddy SR, Elnitski L, Emes RD, Eswara P, Eyras E, Felsenfeld A, Fewell GA, Flicek P, Foley K, Frankel WN, Fulton LA, Fulton RS, Furey TS, Gage D, Gibbs RA, Glusman G, Gnerre S, Goldman N, Goodstadt L, Grafham D, Graves TA, Green ED, Gregory S, Guigo R, Guyer M, Hardison RC, Haussler D, Hayashizaki Y, Hillier LW, Hinrichs A, Hlavina W, Holzer T, Hsu F, Hua A, Hubbard T, Hunt A, Jackson I, Jaffe DB, Johnson LS, Jones M, Jones TA, Joy A, Kamal M, Karlsson EK, Karolchik D, Kasprzyk A, Kawai J, Keibler E, Kells C, Kent WJ, Kirby A, Kolbe DL, Korf I, Kucherlapati RS, Kulbokas EJ, Kulp D, Landers T, Leger JP, Leonard S, Letunic I, Levine R, Li J, Li M, Lloyd C, Lucas S, Ma B, Maglott DR, Mardis ER, Matthews L, Mauceli E, Mayer JH, McCarthy M, McCombie WR, McLaren S, McLay K, McPherson JD, Meldrim J, Meredith B, Mesirov JP, Miller W, Miner TL, Mongin E, Montgomery KT, Morgan M, Mott R, Mullikin JC, Muzny DM, Nash WE, Nelson JO, Nhan MN, Nicol R, Ning Z, Nusbaum C, O'Connor MJ, Okazaki Y, Oliver K, Overton-Larty E, Pachter L, Parra G, Pepin KH, Peterson J, Pevzner P, Plumb R, Pohl CS, Poliakov A, Ponce TC, Ponting CP, Potter S, Quail M, Reymond A, Roe BA, Roskin KM, Rubin EM, Rust AG, Santos R, Sapojnikov V, Schultz B, Schultz J, Schwartz MS, Schwartz S, Scott C, Seaman S, Searle S, Sharpe T, Sheridan A, Shownkeen R, Sims S, Singer JB, Slater G, Smit A, Smith DR, Spencer B, Stabenau A, Stange-Thomann N, Sugnet C, Suyama M, Tesler G, Thompson J, Torrents D, Trevaskis E, Tromp J, Ucla C, Ureta-Vidal A, Vinson JP, Von Niederhausern AC, Wade CM, Wall M, Weber RJ, Weiss RB, Wendl MC, West AP, Wetterstrand K, Wheeler R, Whelan S, Wierzbowski J, Willey D, Williams S, Wilson RK, Winter E, Worley KC, Wyman D, Yang S, Yang SP, Zdobnov EM, Zody MC, Lander ES (2002). Initial sequencing and comparative analysis of the mouse genome. Nature.

[B30] Clark AG, Glanowski S, Nielsen R, Thomas PD, Kejariwal A, Todd MA, Tanenbaum DM, Civello D, Lu F, Murphy B, Ferriera S, Wang G, Zheng X, White TJ, Sninsky JJ, Adams MD, Cargill M (2003). Inferring nonneutral evolution from human-chimp-mouse orthologous gene trios. Science.

[B31] Chen FC, Li WH (2001). Genomic divergences between humans and other hominoids and the effective population size of the common ancestor of humans and chimpanzees. Am J Hum Genet.

[B32] Fujiyama A, Watanabe H, Toyoda A, Taylor TD, Itoh T, Tsai SF, Park HS, Yaspo ML, Lehrach H, Chen Z, Fu G, Saitou N, Osoegawa K, de Jong PJ, Suto Y, Hattori M, Sakaki Y (2002). Construction and analysis of a human-chimpanzee comparative clone map. Science.

[B33] Sachidanandam R, Weissman D, Schmidt SC, Kakol JM, Stein LD, Marth G, Sherry S, Mullikin JC, Mortimore BJ, Willey DL, Hunt SE, Cole CG, Coggill PC, Rice CM, Ning Z, Rogers J, Bentley DR, Kwok PY, Mardis ER, Yeh RT, Schultz B, Cook L, Davenport R, Dante M, Fulton L, Hillier L, Waterston RH, McPherson JD, Gilman B, Schaffner S, Van Etten WJ, Reich D, Higgins J, Daly MJ, Blumenstiel B, Baldwin J, Stange-Thomann N, Zody MC, Linton L, Lander ES, Altshuler D (2001). A map of human genome sequence variation containing 1.42 million single nucleotide polymorphisms. Nature.

[B34] Reich DE, Schaffner SF, Daly MJ, McVean G, Mullikin JC, Higgins JM, Richter DJ, Lander ES, Altshuler D (2002). Human genome sequence variation and the influence of gene history, mutation and recombination. Nat Genet.

[B35] Stringer C (2002). Modern human origins: progress and prospects. Philos Trans R Soc Lond B Biol Sci.

[B36] Iafrate AJ, Feuk L, Rivera MN, Listewnik ML, Donahoe PK, Qi Y, Scherer SW, Lee C (2004). Detection of large-scale variation in the human genome. Nat Genet.

[B37] Carter NP (2004). As normal as normal can be?. Nat Genet.

[B38] Yang Z (1993). Maximum-likelihood estimation of phylogeny from DNA sequences when substitution rates differ over sites. Mol Biol Evol.

[B39] Yang Z (1997). PAML: a program package for phylogenetic analysis by maximum likelihood. Comput Appl Biosci.

[B40] Yang Z, Bielawski JP (2000). Statistical methods for detecting molecular adaptation. Trends Ecol Evol.

[B41] Massingham T, Goldman N (2005). Detecting amino acid sites under positive selection and purifying selection. Genetics.

[B42] Nei M, Gu X, Sitnikova T (1997). Evolution by the birth-and-death process in multigene families of the vertebrate immune system. Proc Natl Acad Sci U S A.

[B43] Liao D (1999). Concerted evolution: molecular mechanism and biological implications. Am J Hum Genet.

[B44] Goriely A, McVean GA, Rojmyr M, Ingemarsson B, Wilkie AO (2003). Evidence for selective advantage of pathogenic FGFR2 mutations in the male germ line. Science.

[B45] Evans PD, Anderson JR, Vallender EJ, Gilbert SL, Malcom CM, Dorus S, Lahn BT (2004). Adaptive evolution of ASPM, a major determinant of cerebral cortical size in humans. Hum Mol Genet.

[B46] Casal J, Gonzalez C, Wandosell F, Avila J, Ripoll P (1990). Abnormal meiotic spindles cause a cascade of defects during spermatogenesis in asp males of Drosophila. Development.

[B47] Riparbelli MG, Massarelli C, Robbins LG, Callaini G (2004). The abnormal spindle protein is required for germ cell mitosis and oocyte differentiation during Drosophila oogenesis. Exp Cell Res.

[B48] Thompson JD, Higgins DG, Gibson TJ (1994). CLUSTAL W: improving the sensitivity of progressive multiple sequence alignment through sequence weighting, position-specific gap penalties and weight matrix choice. Nucleic Acids Res.

[B49] Birney E, Andrews D, Bevan P, Caccamo M, Cameron G, Chen Y, Clarke L, Coates G, Cox T, Cuff J, Curwen V, Cutts T, Down T, Durbin R, Eyras E, Fernandez-Suarez XM, Gane P, Gibbins B, Gilbert J, Hammond M, Hotz H, Iyer V, Kahari A, Jekosch K, Kasprzyk A, Keefe D, Keenan S, Lehvaslaiho H, McVicker G, Melsopp C, Meidl P, Mongin E, Pettett R, Potter S, Proctor G, Rae M, Searle S, Slater G, Smedley D, Smith J, Spooner W, Stabenau A, Stalker J, Storey R, Ureta-Vidal A, Woodwark C, Clamp M, Hubbard T (2004). Ensembl 2004. Nucleic Acids Res.

[B50] Eddy SR (1996). Hidden Markov models. Curr Opin Struct Biol.

[B51] Birney E, Durbin R (2000). Using GeneWise in the Drosophila annotation experiment. Genome Res.

[B52] Morgenstern B (1999). DIALIGN 2: improvement of the segment-to-segment approach to multiple sequence alignment. Bioinformatics.

[B53] Yang Z, Nielsen R, Hasegawa M (1998). Models of amino acid substitution and applications to mitochondrial protein evolution. Mol Biol Evol.

[B54] Goldman N, Yang Z (1994). A codon-based model of nucleotide substitution for protein-coding DNA sequences. Mol Biol Evol.

[B55] Nielsen R, Yang Z (1998). Likelihood models for detecting positively selected amino acid sites and applications to the HIV-1 envelope gene. Genetics.

[B56] Tamura K, Nei M (1993). Estimation of the number of nucleotide substitutions in the control region of mitochondrial DNA in humans and chimpanzees. Mol Biol Evol.

[B57] Xia X, Xie Z (2001). DAMBE: software package for data analysis in molecular biology and evolution. J Hered.

[B58] Letunic I, Copley RR, Schmidt S, Ciccarelli FD, Doerks T, Schultz J, Ponting CP, Bork P (2004). SMART 4.0: towards genomic data integration. Nucleic Acids Res.

[B59] Guex N, Peitsch MC (1997). SWISS-MODEL and the Swiss-PdbViewer: an environment for comparative protein modeling. Electrophoresis.

[B60] Felsenstein J (2004). PHYLIP (Phylogeny Inference Package) version 3.6.

[B61] Page RD (1996). TreeView: an application to display phylogenetic trees on personal computers. Comput Appl Biosci.

[B62] Database of Genomic Variants. http://projects.tcag.ca/variation.

[B63] Sonnhammer EL, Durbin R (1995). A dot-matrix program with dynamic threshold control suited for genomic DNA and protein sequence analysis. Gene.

